# Stupefying Animals before Slaughter

**Published:** 1896-06

**Authors:** 


Stupefying Animals Before Slaughter. Toward the end of last year a law was introduced into the German Reichstag providing for the stupefaction of all animals, excepting poultry, before slaughter. In lieu of the same a committee of the English Royal Society for the prevention of cruelty to animals in slaughtering and the Jewish butcher commission in Manchester have offered a prize of $500 for some preventive means by which an animal can be held fixed and in position for the death-blow or cut. Further conditions can be secured from the secretary, R. S. P. C. A., Manchester, England, Albert Square 9.—*Ibid*, January 15, 1896.

